# Facial EMG Responses to Emotional Expressions Are Related to Emotion Perception Ability

**DOI:** 10.1371/journal.pone.0084053

**Published:** 2014-01-28

**Authors:** Janina Künecke, Andrea Hildebrandt, Guillermo Recio, Werner Sommer, Oliver Wilhelm

**Affiliations:** 1 Department of Psychology, Humboldt Universität zu Berlin, Berlin, Germany; 2 Department of Psychology, University Ulm, Ulm, Germany; The University of Queensland, Australia

## Abstract

Although most people can identify facial expressions of emotions well, they still differ in this ability. According to embodied simulation theories understanding emotions of others is fostered by involuntarily mimicking the perceived expressions, causing a “reactivation” of the corresponding mental state. Some studies suggest automatic facial mimicry during expression viewing; however, findings on the relationship between mimicry and emotion perception abilities are equivocal. The present study investigated individual differences in emotion perception and its relationship to facial muscle responses - recorded with electromyogram (EMG) - in response to emotional facial expressions. *N*° = °269 participants completed multiple tasks measuring face and emotion perception. EMG recordings were taken from a subsample (*N*° = °110) in an independent emotion classification task of short videos displaying six emotions. Confirmatory factor analyses of the *m. corrugator supercilii* in response to angry, happy, sad, and neutral expressions showed that individual differences in *corrugator* activity can be separated into a general response to all faces and an emotion-related response. Structural equation modeling revealed a substantial relationship between the emotion-related response and emotion perception ability, providing evidence for the role of facial muscle activation in emotion perception from an individual differences perspective.

## Introduction

The identification and production of facial expressions of emotion are important interpersonal abilities. Although people normally perform well in classifying facial expressions of basic emotions, there are considerable individual differences in the accuracy of their judgments [1]. Hildebrandt, Sommer, Schacht, and Wilhelm [2] found that individual differences in the ability of perceiving facial expressions of emotion are partially independent of abilities in face perception and general cognition. Here we use the term ability of “perceiving” facial expressions of emotions (or short: emotion perception) instead of emotion recognition which is often used in the literature. The tasks we use to measure this ability include identification (or classification) and visual search. So the ability is captured in a rather broad sense.

It is well documented that perceiving emotional facial expressions of others elicits corresponding facial responses in the observer, which are incidental and covert – often invisible – but measurable by electromyography (EMG; [3], [4]). These responses– often termed facial mimicry – are considered to be automatic as they occur already within 300 ms after stimulus presentation [5]. Previous studies have shown EMG activity in the corresponding muscles during the perception of facial expressions [4]–[6]. For example, the *m. corrugator supercilii* (*corr*) is important for frowning in expressions like anger or sadness, and *m. zygomaticus major* (*zyg*) produces smiling expressions (e.g. [7]). The occurrence and intensity of mimicry are modulated by stimulus type, task, and context [8]–[11]. Facial mimicry is more pronounced in response to dynamic than static facial expressions (e.g., [12]).

According to embodied simulation theories, involuntarily mimicking emotional expressions of others fosters the understanding of these emotions by simulating of the corresponding mental states in the perceiver [13]–[16]. Simulation in this sense implies the reactivation of basal motoric, somatosensory, affective and reward-related systems representing the meanings of the perceiver's expressions [17]. Hence, perceiving an emotional facial expression supposedly triggers simulation, that is, the perceiver uses his bodily and neural states elicited by the perceived expression [16] in order to access the corresponding emotional concept. Covert facial mimicry is attributed to sub-threshold muscular simulation of emotions [14].

If simulation is indeed a fundamental aspect of emotion processing, facial mimicry should be a functional element of emotion-related abilities. Thus, the intensity of facial mimicry should be related to the accuracy of emotion perception (EP). In line with this view, experimental studies have shown that obstructing incidental mimicry leads to performance impairment in EP [18], [19].

In contrast to these reports Hess and colleagues did not observe a correlation between incidental mimicry and EP [6], [20]. A relationship between anger perception and anger mimicry was only present for elderly but not for younger participants [21].

In a recent review Hess and Fischer [22] concluded that facial mimicry is not necessary for the classification of prototypical emotional displays. In their *Emotion Mimicry in Context* view they argue that emotional mimicry requires a specific context in which signals are interpreted as emotional intentions. Moreover, empirical evidence of covert facial mimicry in response to simple facial expression stimuli speaks for valence-related facial responses rather than for emotion-specific mimicry [22]. This is in line with the view that facial responses of *corr* and *zyg* reflect affective states in the perceiver. Larsen, Norris, and Cacioppo [23] reported a substantial relation between facial muscle activity and self-reported valence ratings in response to affective pictures, words, and sounds. These affect-related facial responses seem to be involuntary or incidental [24]. If incidental facial responses to emotional stimuli reflect an affective state in the perceiver, perception of emotional expressions could lead to a simulation of an affective state which facilitates the access to emotional concepts in the same way as emotion-specific mimicry could.

In sum, evidence regarding the relation between incidental facial responses to facial expressions of emotions - in terms of emotion-specific or valence-related simulation - and EP is inconclusive. Testing the relationship between individual differences in the amount of facial responses to emotional expressions and EP ability within an individual differences approach is therefore of pivotal interest for embodied simulation theories and may provide new insights on the role of mimicry in EP.

It is important to separate construct-related variance from method specificity of the behavioral indicators and their measurement error (e.g., [25].). This can be accomplished by using a broader variety of tasks to measure a construct of interest. By establishing a latent variable that captures the communality of the behavioral indicators collected with those tasks it is possible to abstract from method specificity and measurement error. Modeling the relationships between latent variables and behavioral indicators also allows deriving estimates of construct reliability and the reliability of its indicators [26].In the present study, we aim to provide more conclusive evidence about the nature and strength of the relationship between facial responses to emotional expressions and EP from an individual differences perspective. To this end we used multivariate assessment and modeling of EP, face perception (FP) and – for the first time in the literature – of facial responses to emotional expressions.

We assessed individual differences in EP and FP with the extensive task battery developed by Herzmann, Danthiir, Schacht, Sommer, and Wilhelm [27] and Wilhelm, Hildebrandt, Manske, Schacht, and Sommer [28]. Incidental facial responses were measured with EMG during a separate emotion classification task. Stimuli were video clips of facial expressions of different intensities, approximating the appearance of expressions in social communication (e.g., [29]). The videos showed six emotions (anger, disgust, fear, happiness, sadness, surprise), and two neutral facial movements [30], allowing to compare different emotions and to separate emotion-related from general responses to face stimuli. Importantly, we heeded experimental independence of all measurements, a crucial aspect when applying correlation techniques.

As a first main aim, we establish a measurement model of incidental covert facial responses to emotional expressions controlling for measurement error in the physiological indicators. The second aim addressed the relationship between EP and facial responses to emotional expressions

Regarding our first aim, we expected stronger EMG activation in *corr* in response to anger and sadness and decreased activation in response to expressions of happiness. Conversely, activation in *zyg* should be stronger for smiles relative to all other expressions (e.g., [4], [23], [31]). Since facial muscles are responsive to all stimuli, effort or cognitive load [32], [33], we also modeled a general face response factor, accounting for the common variance of EMG indicators for both emotional and neutral facial expressions. We predicted that the average EMG activity in emotion-relevant muscles should reliably indicate a latent emotional facial response factor. Such a general emotion-related factor is theoretically plausible; however, personality traits and exposure effects might lead to emotion-specific rank orders of individuals in facial responses to emotional expression. For example, neuroticism or depressiveness might specifically affect facial responses to anger and sadness [34]. Thus, we postulated emotion-specific factors ordered under the general face response factor.

Regarding our second aim, and in line with embodied simulation theories we expected a substantial correlation between emotion-related facial responses and EP. The ability to correctly perceive and recognize unfamiliar faces is highly correlated with EP and therefore a highly relevant covariate [2]. By controlling for FP in EP on the behavioral side and general muscular responses to face stimuli in facial responses to emotional expressions on the physiological level we aimed to rule out alternative, more general explanations for a possible relationship, for instance attention or reactivity to face stimuli per se. Such a multivariate individual differences approach constitutes a new perspective on testing the relationship of facial responses to emotional expressions with EP.

## Materials and Methods

### Ethics Statement

This study received institutional ethics approval (provided from the committee of ethics of the Department of Psychology, Humboldt-University Berlin). Written informed consent was obtained from all participants included in the study reported here.

### Design and Sample

The study consisted of two parts, a psychometric and a psychophysiological one. In the psychometric part 269 participants (52.4% female) completed three FP and three EP tasks. Additionally, they completed measures of object cognition and general cognitive abilities, which are not in the scope of this article and will be reported elsewhere. Mean age was 25.90 years (*SD*° = °4.41), educational background was heterogeneous (21.1% without, 48.0% with high school degrees, and 30.8% with academic degrees), and all participants reported normal or corrected-to-normal visual acuity; 175 of them volunteered for psychophysiological part as well. Of those volunteers a subsample of 110 participants (45.5% female) was randomly selected. This subsample was representative of the total sample regarding age (*M*° = °26.55, *SD* = 4.82) and educational background (47.3% and 27.3% with high school and academic degrees, respectively; 25.4% without degree). The psychophysiological part consisted of three tasks (a face familiarity test, an emotion classification task, and an explicit emotion expression task) in which we co-registered EEG and EMG. Here, we only report the EMG data recorded during the emotion classification task. The EEG data deal with emotion-related ERP components while recognizing dynamic facial expressions of emotions and will be reported elsewhere [30].

### Stimuli and Procedure

#### Psychometric part

FP and EP tasks will be shortly described in the following. All stimuli were gray-scaled, adjusted in skin texture and fitted with standardized head-size into a vertical elliptical frame of 200×300 pixel. We refer to Herzmann, Danthiir, Schacht, Sommer, & Wilhelm [27] for more details on the task procedures and examples of stimuli for the face cognition tasks. Stimulus examples and schematic representations of the EP tasks are provided in the supporting information (Figures S1 and S2).

1. *Sequential Matching of Part–Whole Faces— with condition Part and condition Whole.* First, a face stimulus was presented for 1000 ms; following a blank screen of 200 ms either two faces (whole condition) or two parts of a face (two pairs of eyes, two noses, two mouths; part condition) were presented. One of these faces or parts of faces was the face or part seen before (target) and the other was a new face or face part (distracter). Participants were expected to identify the target. Part and whole conditions served as different indicators.
2. *Simultaneous Matching of Spatially Manipulated Faces—with Conditions of Upright and Inverted.* Participants had to decide if two faces presented simultaneously were the same or different. Faces were generated from identical pictures by changing spatial relations between features (e.g., distance between eyes and nose, between the eyes, or between mouth and nose). In 50% of the trials faces were different. Moreover, in 50% of the trials both faces were presented upside down (inverted). Upright and inverted conditions were defined as separate indicators.
3. *Facial Resemblance.* In each trial three faces were presented simultaneously. Participants had to indicate, which of two morphed faces in frontal view most resembled a third face shown in three-quarter view (target face). Both morphs consisted of a mixture of the target face and another unfamiliar face (e.g., 20% face A and 80% face B, for morph 1; 40% face A and 60% face B, for morph 2; face B being the target face).
4. *Identification of Emotion Expressions from Composite Faces.* Participants saw composite faces of the same person displaying different emotions in the upper and lower halves of the face. The composition of the emotion was constructed according to empirical findings that some emotional expressions are better recognized from the upper part of the face, and others from the lower [35]. There were nine different expression composites: Anger/disgust, anger/happiness, anger/surprise, fear/disgust, fear/happiness, fear/surprise, sadness/disgust, sadness/happiness and sadness/surprise. Composites were presented with a prompt word (“TOP” or “BOTTOM”). Participants indicated the emotion expressed in the face half indicated by the prompt word by pressing one of six emotion-labeled buttons. Unbiased hit rates [36] were defined as indicators.
5. *Identification of Emotion Expressions of Different Intensity from Upright and Inverted Dynamic Face Stimuli.* Stimuli were video clips of six dynamic emotional expressions developing within 500 ms. Emotional expressions were morphed to reach three different intensity levels (i.e., mixing neutral and emotional expression to different degrees: 20% neutral and 80% emotional, 40% neutral and 60% emotional, 80% neutral and 20% emotional); faces were presented either upright or inverted (50% of the trials each). The frame displaying the full emotional expression remained on the screen until participants made a classification response. We used unbiased hit rates [36] as performance indicators.
6. *Visual Search for Faces with Corresponding Emotion Expressions of Different Intensity.* Nine face stimuli of the same person were presented simultaneously in a 3×3 grid. The majority of pictures displayed the same emotion, but a varying number of targets (1, 2, 3 or 4) showed a neutral or a different emotional expression. In this paradigm the emotions of anger, disgust, fear, sadness, and surprise were used. Participants had to identify and indicate the expressions that diverged from the dominating emotion by setting check marks via mouse click below the corresponding stimuli. The number of targets to be detected was indicated by a number within a box on top of the screen.

The order of these six tasks during the psychometric part was the same for all participants (1, 3, 4, 5, 2, and 6). The FP and EP tasks were interspersed between other tasks measuring object cognition and general cognitive abilities.

#### Psychophysiological part

**Figure 1:**
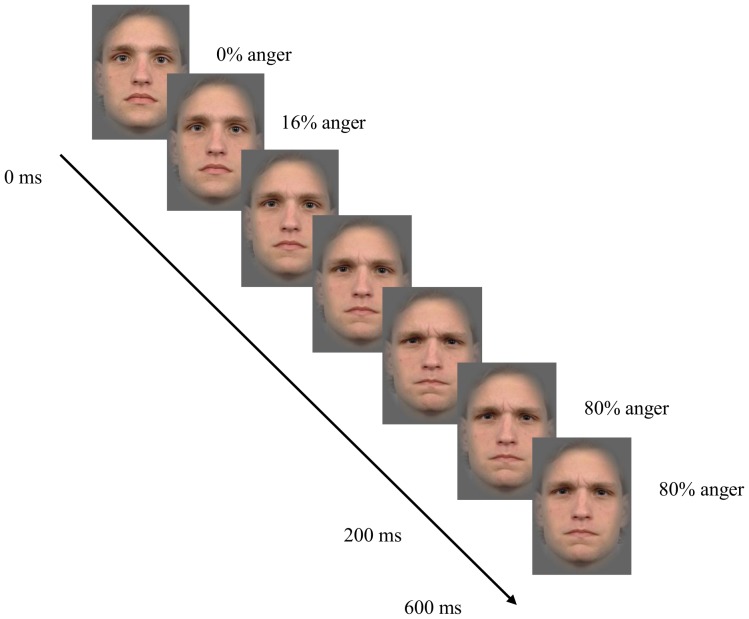
Time course of development of facial expression display within the first 6 frames (0–200 ms) of a trial; the last 12 frames (200–600 ms) showed full emotional expressions.

Stimuli were color videos of six facial expressions (anger, disgust, fear, happiness, sadness, surprise) displayed at two intensity levels (100%, 80%), and two types of neutral movements (blinking, chewing). All stimuli were taken from the Radboud Faces Database [37] and were morphed with FantaMorph© software [38]. The video frames (30 fps) increased linearly in expression intensity from neutral to either 100% or 80% expression within 200 ms, hence, in a sequence of six frames. The last frame with the most intense emotional expression was displayed for another 400 ms. All faces were 8×12 cm in size and framed by a gray oval (see Fig. 1).

Participants sat in an electrically shielded cabin with dimmed lighting. Viewing distance to a 17 inch LCD computer-monitor (75 Hz refresh rate, 1280×1024 pixels) was 80 cm. Trials started with a fixation cross presented for 700 ms, followed by a (video) face stimulus displayed for 600 ms, on a background of the same light gray color as the oval frame. In each trial participants categorized the expression of the face stimulus by mouse-clicking one of seven boxes labeled with the German words for the six basic emotions and “neutral”. The spatial arrangement of the labels was kept constant across trials and participants. The scale was present until a response occurred. After the response a blank screen of 500 ms was shown, followed by the next trial. The reader may have noticed that the presentation time of 600 ms and the inter-stimulus-interval employed here were shorter than in other EMG studies. This was done in order to optimize the study design for co-registration with EEG which requires more repetitions trials. Recio, Schacht, and Sommer [39] showed that ISI variation while co-registration of SCR (a comparable slow peripheral signal) and EEG did not affect the effects in general.

The set of trials included portraits of 38 models displaying all seven expressions at both intensity levels; all models were shown once in both intensity levels; a third presentation occurred in the low intensity condition for 50% of the models and for the others in the high intensity condition. This resulted in a total of 798 trials, 57 for each of the 14 conditions; order of presentation was randomized, with short, self-administered breaks after every 200 trials. The psychophysiological experiment reported here, followed a face familiarity task of 60 min and a 10-min break. It took 45 min and was followed after another break by an explicit expression task (see below). During the whole session participants were monitored by means of a non-recording video camera.

### EMG Recordings

Facial EMG was measured with two pairs of Ag/AgCl electrodes, 4 mm in diameter, one each for the *corr*, and *zyg* muscle, placed on the left side of the face, as recommended by Fridlund and Cacioppo [40]; ground was placed on the upper half of the right forehead [41]. Impedances were kept below 10 kΩ using a conductive EMG-gel (Neurgel). The raw EMG signal was amplified and filtered online at a band pass of 8–10000 Hz, using a Coulbourn V75-04 bio-amplifier and rectified and integrated with a Coulbourn V76-24 contour following integrator (TC° = °10 ms). The signal was digitized with BrainVision Recorder Software (Brain Products GmbH, 2010) at a sampling rate of 1000 Hz and notch-filter at 50 Hz.

### Data Analyses

#### Psychometric data

Performance indicators from the psychometric part were the average number of correctly solved trials. For two of the EP tasks with categorical judgments unbiased hit rates were calculated [36] and used as indicators for the measurement model. These scores control for response bias, which often occurs in emotion classification tasks since some emotions are typically confused with each other. In order to detect multivariate outliers, we calculated Mahalanobis Distances in Mplus 5.21 [42]. There were no severe outliers detected by visual inspection.

#### EMG data

Five participants were excluded due to error rates exceeding 25%. Further participants were excluded due to EMG data quality as follows. After the emotion classification task, participants completed an explicit emotion expression task. In this task subjects were asked to produce angry and happy facial expressions. The EMG signals recorded during this task served as sensitivity check for the EMG. Thus, exclusion of participants for a given EMG data set in the emotion classification task was based on the EMG activity during the explicit emotion expression task. EMG data sets for a given muscle and participant were discarded if there was no discernible EMG activity of that muscle during the explicit task. This led to a further exclusion of 10 participants based on *corr* data and of 36 on *zyg* data. The high exclusion rate for *zyg* data might be due to the high variability of the morphology of this muscle [43]. This is no exception; for example, Aguado, Román, Rodríguez, Diéguez-Risco, Romero-Ferreiro et al. [44] reported that only in 58% of their participants *zyg* activity differentiated between emotions.

The EMG signal was segmented offline into 1200-ms epochs, including a 200-ms pre-stimulus baseline. This time-window appears to be sufficient to detect peaks of *corr* and *zyg* activity in response to dynamic facial expressions [12]. For each muscle and participant we performed an automatic artifact rejection implemented in MATLAB R2010a based on the *SD* of EMG over all trials of a given participant. If the range within a 50-ms segment in a given trial exceeded 3 *SD*s, the trial was rejected. This automatic method was compared to manual artifact rejection and delivered highly similar results. Even blink artifacts were identified with this algorithm.

We also rejected all trials with incorrect responses. Remaining trials were *z*-standardized within each participant (e.g., [6]). On average we rejected 8.50% of trials for *corr* and 2.60% for *zyg*. In order to extract reliable averaged EMG responses, we required at least 18 averaged trials for each indicator. One additional participant of the *corr* sample was excluded due to this criterion. Final analyses of *corr* and *zyg* data were performed for *N* = 94 and 69 participants, respectively. There were no multivariate outliers for EMG data according to the same type of inspection as for the psychometric data. Finally, we calculated average EMG activities for the 200-ms pre-stimulus baseline and 10 consecutive 100-ms segments starting at stimulus onset.

#### Statistical analyses

Experimental effects on the average EMG activity were tested with repeated-measures analysis of variance (rmANOVA) with factors expression (7 levels) and intensity (2 levels). Given the different nature of the manipulation in intensity for emotional and neutral expressions, the assignment of the blinking and chewing conditions to high and low intensity levels were defined according the muscle. For *corr* blinking was defined as high intensity condition assuming that motion is more salient in the eye. Conversely for *zyg*, chewing was defined as high intensity. If the sphericity assumption was violated, *p*-values corresponding to Greenhouse-Geisser adjusted degrees of freedom are reported. All post-hoc comparisons were Bonferroni corrected. Given the large sample size, we interpret effect sizes partial η*^2^*(η_p_^2^)°>°0.15 rather than *p*-values.

For confirmatory factor analyses (CFA) and structural equation modeling (SEM) [45] the trials of each condition (7 expressions×2 intensities) were split into odd and even, generating a total of 28 conditions. The averaged mean EMG activity in the segments from 300 to 800 ms in the emotional and neutral conditions were used as indicators in CFA and SEM. Latent variable analyses were conducted using the *lavaan* package [46] implemented in the R Environment for Statistical Computing.

## Results

### Experimental Effects

**Figure 2:**
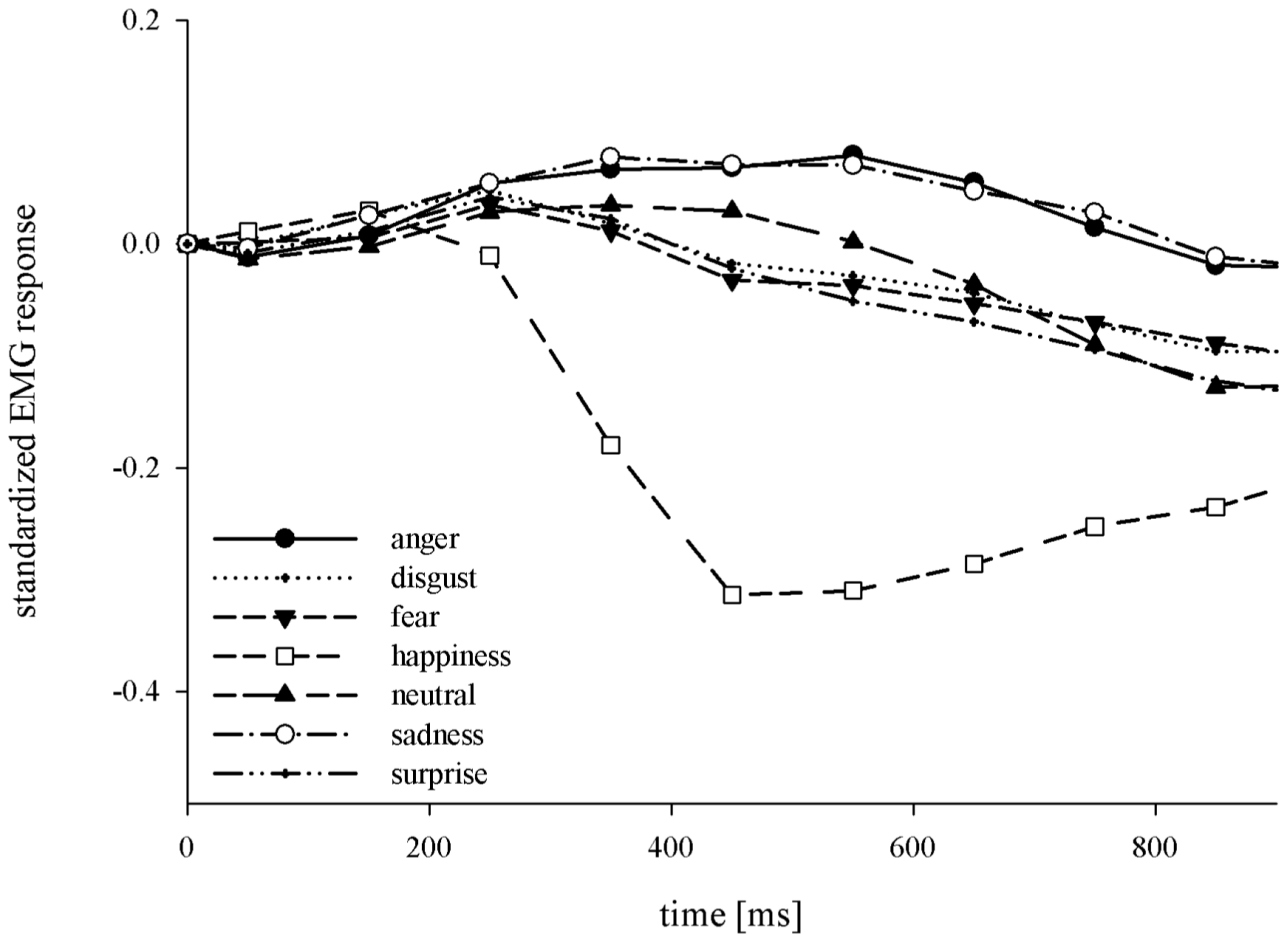
Time course of *corr* z-standardized EMG response for all emotion conditions.

For the *corr* activity, the rmANOVA in consecutive 100-ms segments showed substantial (*p*°<°.05, η_p_ *^2^*°>°0.15) effects of emotion starting at 300 ms, *F*(2.90, 269.90)° = °44.31, *p*°<°.001, η_p_ *^2^*° = °.323, continuing with a similar effect size (*ps*°<°.05, η_p_ *^2^s*°>°.214) until 900 ms. Post-hoc analyses indicated that the *corr* activity in response to happy faces was diminished (*ps*°<°.05) relative to all other emotions and to the neutral conditions in all segments between 300–900 ms. Significantly higher *corr* activity for angry and sad expressions started in the 500–600 ms segment and lasted until 900–1000 ms (*ps*°<°.05). Figure 2 shows the mean amplitude of *corr* activity for all emotions over time. There were significant effects for intensity from 0–300 ms, but effect sizes were small, *F*s(1, 93)°<°11.84, *p*s°≥°.001 and the η_p_ *^2^*values were°<°.113. The emotion x intensity interaction reached significance at 500–600 ms but the effect size was very small, *F*(6, 558)° = °2.33, *p*° = °.031, η_p_ *^2^*° = °.024.

The *zyg* data did not reveal any significant experimental effects. Table 1 shows *p*-values and effects sizes for main and interaction effects for all time-windows for both muscles. Detailed tables with descriptive statistics for accuracy of emotion classification and means, standard deviations and standard errors for all time windows for *corr* and *zyg* are provided in the supporting information (see Tables S1 and S2).

**Table 1:** *p*-values (two-tailed) and partial η^2^ for main effect of emotion (E) and intensity (I) and interaction effects (E×I) for all time windows for *corr* and *zyg*.

		0–100		100–200		200–300		300–400		400–500		500–600		600–700		700–800		800–900		900–1000	
		*p*	η_p_^2^	*p*	η_p_^2^	*p*	η_p_^2^	*p*	η_p_^2^	*p*	η_p_^2^	*p*	η_p_^2^	*p*	η_p_^2^	*p*	η_p_^2^	*p*	η_p_^2^	*p*	η_p_^2^
*corr*	E	.173	.016	.038	.024	<.001	.068	<.001	**.323**	<.001	**.379**	<.001	**.376**	<.001	**.338**	<.001	**.269**	<.001	**.214**	<.001	**.173**
	I	.021	.056	.001	.113	.006	.080	.192	.018	.183	.019	.145	.023	.104	.028	.204	.017	.889	<.001	.626	.003
	E×I	.919	.004	.720	.007	.067	.021	.187	.016	.073	.020	.031*	.024	.156	.017	.102	.019	.472	.010	.441	.010
*zyg*	E	.737	.009	.388	.015	.501	.013	.405	.015	.456	.014	.808	.007	.487	.013	.318	.017	.217	.020	.166	.022
	I	.506	.007	.547	.005	.820	.001	.975	<.001	.951	<.001	.531	.006	.317	.015	.530	.006	.863	<.001	.828	.001
	E×I	.240	.019	.302	.017	.178	.022	.308	.017	.154	.023	.198	.021	.369	.016	.751	.008	.718	.009	.504	.013

effect sizes η_p_^2^>.15 are in bold.

**Figure 3:**
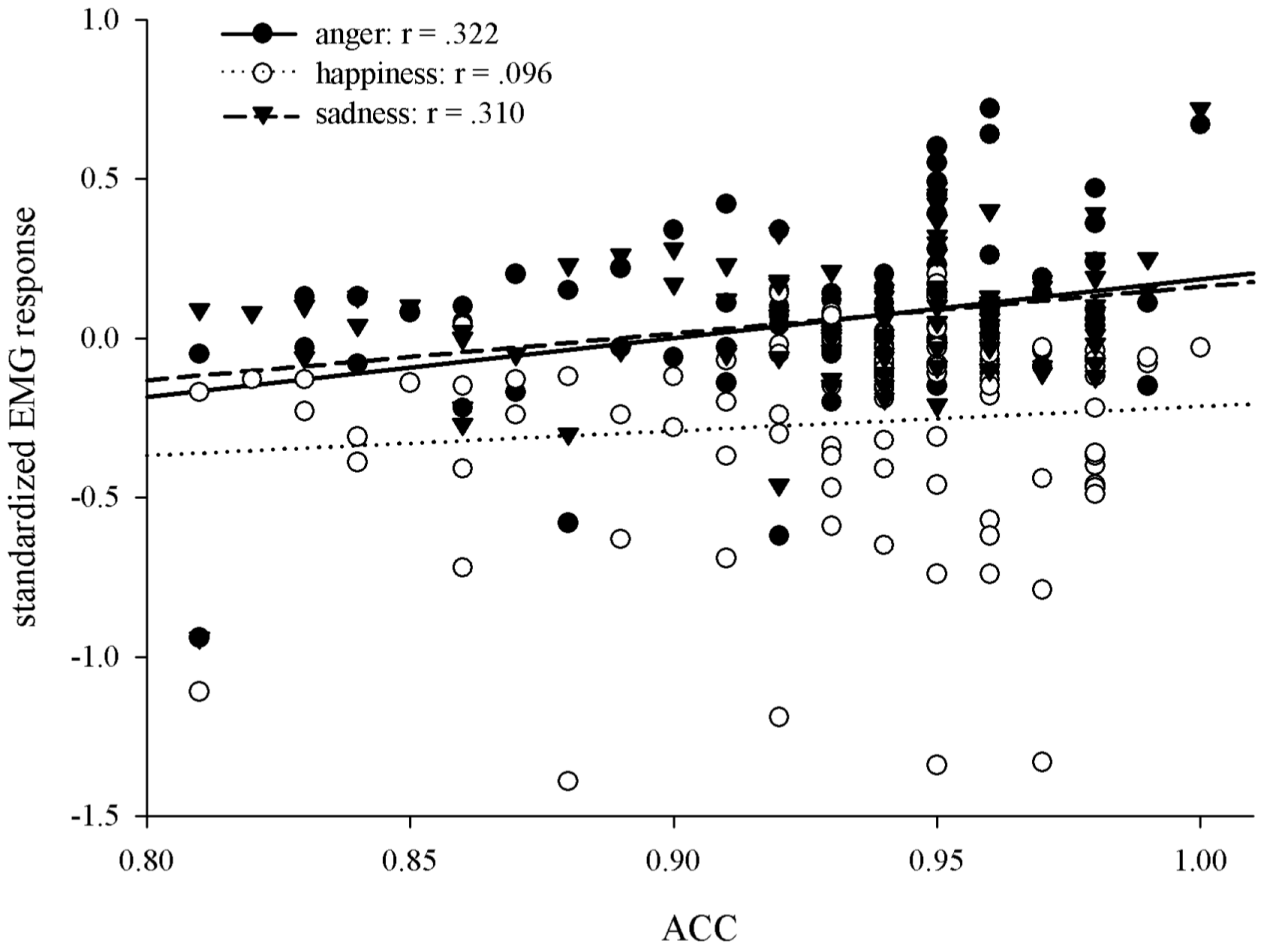
Correlations between mean accuracy rates and mean *corr* actvitiy (300–800 ms) to anger, happiness and sadness within the EMG emotion classification task.

Since *zyg* data showed no emotion-related effects, only data from *corr* were considered for further analyses. We analyzed the correlation between the mean *corr* activity and accuracy rates for the conditions where experimental effects were substantial (mean *corr* activity in response to angry, happy, and sad facial expressions in the 300–800 ms time-window). This analysis of *corr* activity and EP accuracy within the EMG emotion classification task revealed significant correlations between the average emotion classification accuracy and the *corr* activity in response to angry (*r*° = °.322, *p*°<°.05) and sad (*r*° = °.310, *p*°<°.05) faces (see Fig. 3). The correlation with *corr* activity in response to happy faces was not significant (*r* = .096, *p* = .356). However, these relationships may be diminished by measurement error. Alternatively, they could be driven by the experimental dependency of the measures as these were assessed within the same trials. Using structural equation modeling these issues will be addressed with the analyses reported further below.

### Measurement Model

For the measurement models for *corr* we only used data from expressions with significant experimental effects on *corr* activity, namely, anger, happiness, sadness, and neutral. Due to the lack of experimental emotion effects in *zyg* recordings, we did not model data from *zyg* EMG. Structural Equation Models were estimated with the maximum-likelihood (ML) algorithm. We report the chi-square test (χ2) that allows estimation of the exact model fit. In addition to χ2, which is sensitive for large sample sizes common for CFA, we report further fit indices: (1) The Comparative Fit Index (CFI) with values above .95 denoting acceptable fit (2) the Root Mean Square Error of Approximation (RMSEA), which should be below .08, and (3) the Standardized Root Mean Square Residual (SRMR), which should not exceed .08 [47]. For comparisons between nested models we used the chi-square difference test (Δχ2). This test is significant (*p*°<°.05) if the restrictions in the nested model lead to substantial decrement of fit compared to the less restricted model. The reliability of latent factors was calculated with the omega (ω) index, which represents the common variance of all indicators of a given factor [48].

In Model 1 both emotional and neutral indicators loaded on a general factor of *corr* responses to faces (Fcorr) and all emotional indicators loaded on an emotion-related factor of *corr* response (Ecorr). The fit of Model 1 was acceptable (χ2 [92]° = °145.63, *p*°<°.001; CFI° = °.959; RMSEA° = °.079; SRMR° = °.036). Model 2 estimated three correlated emotion category-related *corr* factors: anger (ANcorr), happiness (HAcorr), and sadness (SAcorr) and Fcorr. Emotion specific *corr* factors accounted for the specific variance in corresponding emotion indicators only. Descriptively, Model 2 fitted the data better than Model 1: χ2 [89]° = °117.98, *p*° = °.022; CFI° = °.978; RMSEA° = °.059; SRMR° = °.031. The correlations between the emotion-specific *corr* factors were substantial (*r*_an/ha_° = °−.543; *p*°<°.001; *r*_an/sa_° = °.741; *p*°<°.001; *r*_ha/sa_° = °−.656, *p*°<°.001). Given these sufficiently high correlations, we included an emotion-related second-order factor (EScorr), accounting for the common variance of ANcorr, HAcorr, and SAcorr (Model 3a). The model fit was the same as in Model 2 because both models are equivalent in terms of the model implied covariance matrix. All factor loadings on Fcorr were similarly high – independent of the emotion category. Thus, it appeared reasonable to restrict the loadings on Fcorr to be equal in a Model 3b. The model fit was still acceptable under this restriction (χ2 [104]° = °137.16, *p*° = °.016; CFI° = °.975; RMSEA° = °.058; SRMR° = °.090). The Δχ2 with 19.175 to 15 Δ*df* was not significant (*p*° = °.206), indicating that the fit was not substantially affected by introducing equality constraints on those loadings. Thus, in all indicators the same amount of variance was accounted for by Fcorr. Moreover, these restrictions increased the robustness of the model, given the relatively small sample size and number of parameters estimated.

**Figure 4:**
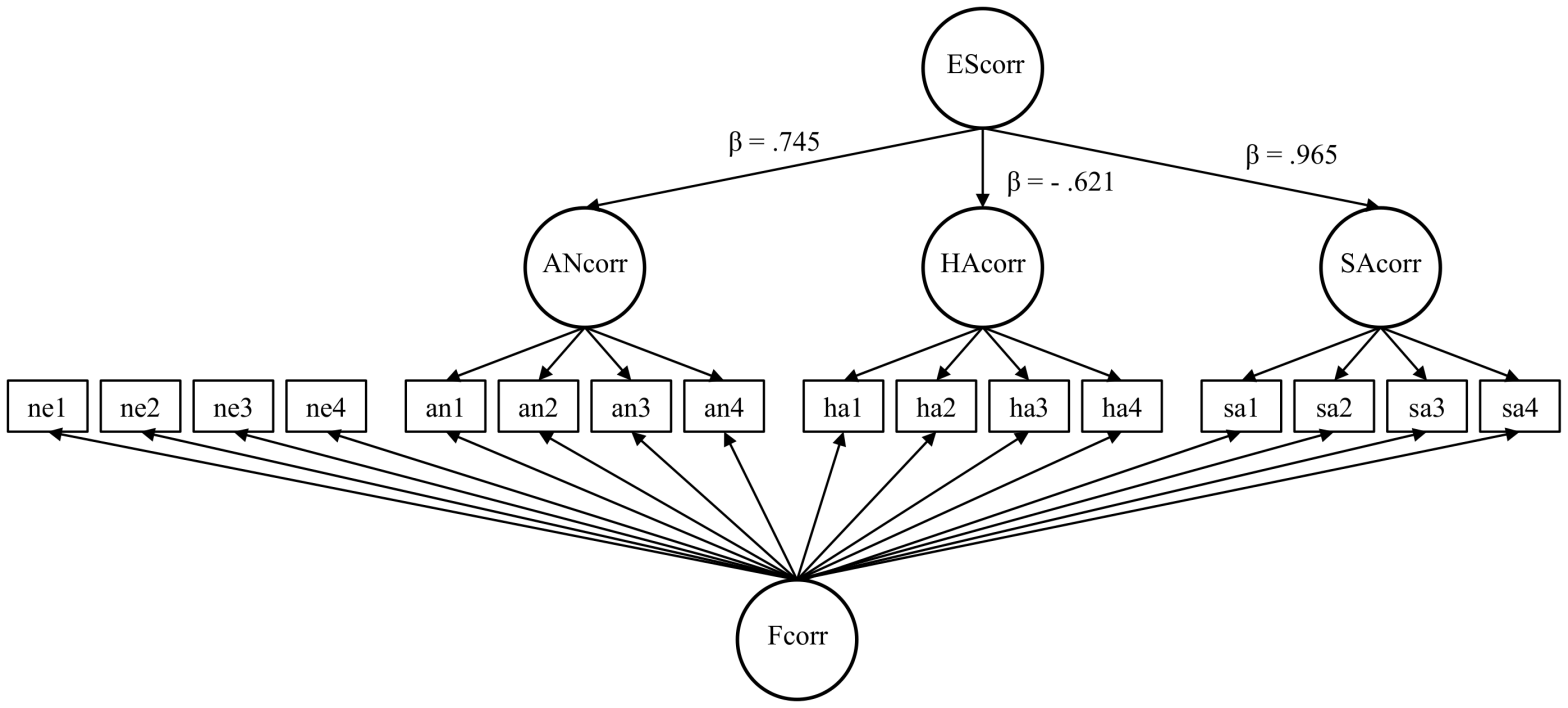
Schematic representation of the measurement model of emotion-related *corr* responses – Model 3b. EScorr = second order emotion-related *corr* factor; ANcorr, HAcorr, SAcorr = *corr* factors as responses to anger, happiness, and sadness, respectively; Fcorr = general face response factor; ne1-n4 = neutral indicators, an1–an4 = anger indicators, ha1–ha4 = happiness indicators, sa1–sa4 = sadness indicators.

The reliabilities of the latent factors were acceptable (ω° = °.936 [Fcorr], ω° = °.627 [ANcorr], ω° = °.799 [HAcorr], ω° = °.338 [SAcorr], and ω° = °.626 [EScorr]). We used Model 3b for the following SEM analyses testing the relationship of facial mimicry with FP and EP (see Fig. 4). Standardized factor loadings estimated in Model 3b can be found in the supporting information (see Table S3).

### Structural Models

**Figure 5:**
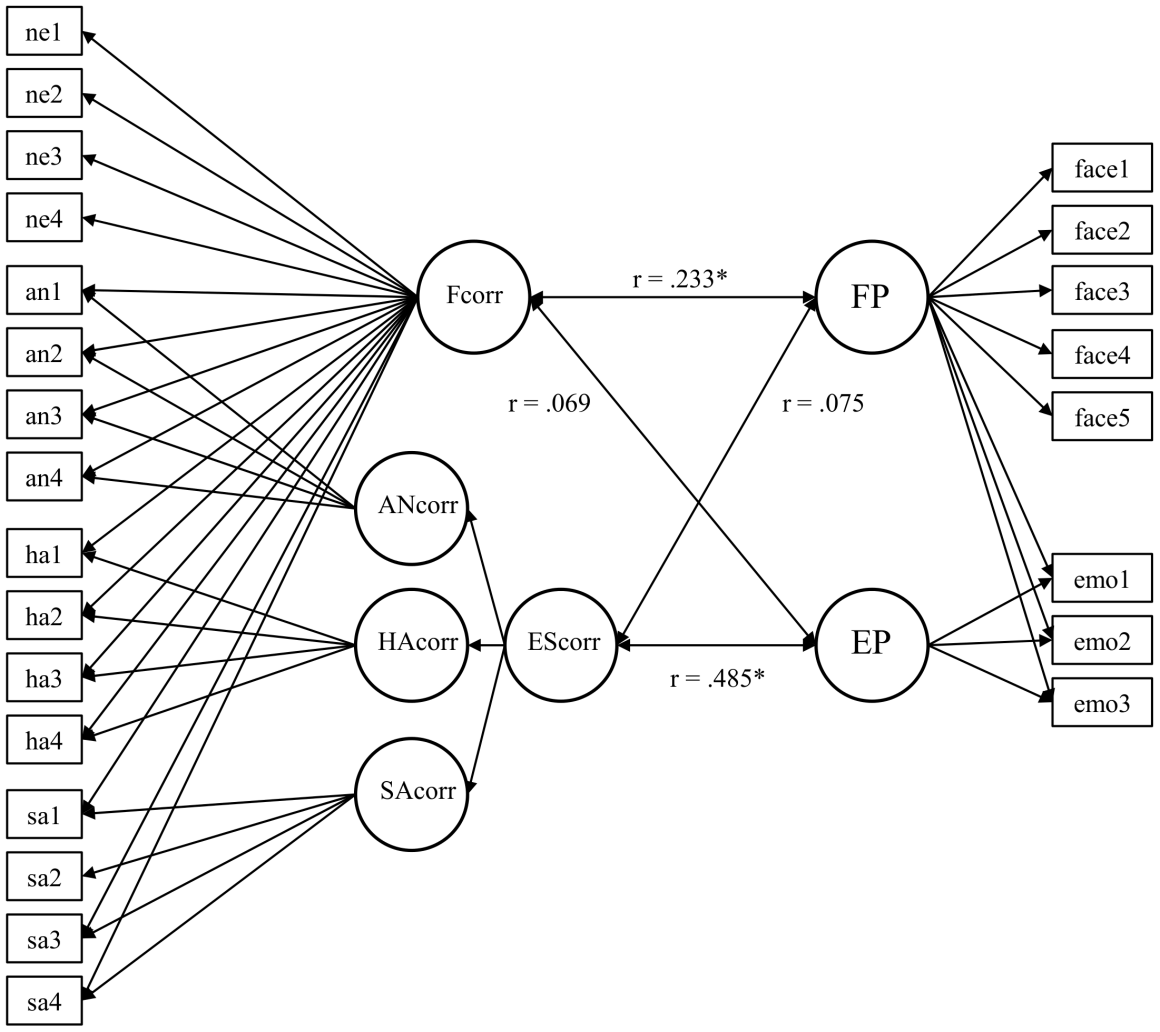
Schematic representation of the structural model of face reaction, emotion-related *corr* response, face perception and emotion perception – Model 1a. EScorr = second order emotion-related *corr* factor; ANcorr, HAcorr, SAcorr = *corr* factors as responses to anger, happiness, and sadness, respectively; Fcorr = general face response factor, ne1 - n4 = neutral indicators, an1–an4 = anger indicators, ha1–ha4 = happiness indicators, sa1–sa4 = sadness indicators, FP = face perception, EP = emotion perception, face1–face5° = °face perception indicators, emo1–emo3 = emotion perception indicators. *p°<°.05, two-tailed.

Next we tested the relationships among Fcorr, EScorr, FP, and EP. There were five FP indicators (face1 to face5) derived from three FP tasks and three EP indicators derived from three EP tasks (emo1, emo2, and emo3). EP was nested under FP; thus FP accounted for the FP-specific variance in all psychometric indicators, while EP captured the specific variance of EP indicators (for factor loadings see supplementary Table S4) after FP was accounted for. In Structural Model 1a all correlations among latent factors were freely estimated (Fig. 5). This model fitted the data well (χ2 [245]° = °321.01, *p*° = °.001; CFI° = °.949; RMSEA° = °.057; SRMR° = °.087). Correlations among FP and EScorr (*r°* = °.075, *p*° = °.585) and EP and Fcorr (*r*° = °.069, *p*° = °.690) were not significant. Significant correlations were observed between FP and Fcorr (*r*° = °.233, *p*° = °.047) and between EP and EScorr (*r*° = °.485, *p*° = °.015). To test if the numerically higher correlation between EP and EScorr compared to FP and EScorr was statistically significant, we estimated a further Structural Model 1b, in which these correlations were constrained to equality. This model showed acceptable fit (χ2 [246]° = °326.31, *p*° = °.001; CFI° = °.946; RMSEA° = °.059; SRMR° = °.090), but the chi-square difference of Δχ2° = °5.3 with Δ*df*° = °1 was significant (*p*° = °.021). Thus, Structural Model 1b with correlations forced to equality fitted worse than the Structural Model 1a with freely estimated correlations, demonstrating that the relationship between EP and EScorr was substantially higher than the correlation between FP and EScorr.

## Discussion

The first aim of the present study was measuring facial responses to emotional expressions as a latent construct by applying measurement models to EMG activity recorded in two facial muscles. Second, we assessed whether individual differences in facial reactions to emotional expressions are substantially related to emotion perception.

In the *corr* muscle facial expressions were mimicked as expected: *Corr* activity increased in response to angry and sad faces and decreased to happy faces. Hypotheses related to the *zyg* muscles could not be confirmed. Accuracy rates in emotion classification correlated with the amount of mimicry in *corr* in response to angry and sad faces within the same experiment. Further, we could establish a measurement model of *corr* responses to emotional expressions; the model involved a general face response factor and an emotion-related second-order *corr* factor that was sensitive for emotions, but integrated across all emotion categories – hence, it was unspecific for any of the three particular emotions. In structural equation modeling including a latent face perception factor and a nested emotion perception factor we found a substantial correlation between emotion-related *corr* response and emotion perception.

In contrast to other studies that examined the correlation between facial mimicry and emotion [6], [20], [21], we found a substantial relationship, at least for the *corr* response to angry, happy and sad faces with EP. This discrepancy might be due to methodological differences of the present study and more traditional single task approaches. In the present study a test battery captured task-general abilities of face and emotion perception, latent variables took into account measurement error, and relationships among construct variances were assessed with structural equation models. In addition, in our structural models we took into account the emotion-unrelated factors of face perception ability and EMG responses to faces in general. These methodological advances may have revealed relationships that remain hidden in single task studies.

According to embodied simulation theories the perception of an emotional expression leads to the simulation of a corresponding affective state in the perceiver which in turn facilitates the access to the emotional concept. Our results suggest that humans show individual differences in facial responses to emotional facial expressions - as indicated by emotion-related *corr* response. This simulation may facilitate the classification of facial expressions through the activation of corresponding emotional concepts. As suggested by Hess and Fischer [22]) this may especially be the case when emotion recognition is difficult - as in the psychometric tasks of the present study.

The observed positive correlation between emotion-elicited *corr* responses and psychometric emotion perception by itself cannot demonstrate causality. However, considering that experimental studies have shown impairments of emotion recognition when facial mimicry is blocked [18], [19], our results provide convergent evidence that the amount of facial responses to emotional expressions is related to emotion perception. Hence, individual differences in emotion-related *corr* responses may– at least to some extent – account for differences in emotion perception ability.

Alternatively one might argue that the differences in *corr* activity between emotion conditions reflect the relatively increased mental effort in more difficult conditions [49]. Since happy faces are easy to identify (accuracy rates are .99) less mental effort is required relative to all other emotional expressions. Thus, the observed relaxation in the *corr* activity in response to happy faces might be due to reduced deployment of processing effort. The relation between emotion-related *corr* response and emotion perception could then be explained by individual differences in mental effort or resource mobilization [50]. However, the mental effort account does not seem to be a plausible explanation of the present *corr* results. Accuracy rates for neutral faces were also relatively high (.96/.97) whereas *corr* activity for neutral faces was as high as for disgust expressions, which showed clearly lower accuracy rates (.85/.84). Moreover, more recent studies did not find a relation between *corr* activity and required mental effort induced through manipulating processing difficulty [51], [52]. Other authors who found a decline in *corr* activity in response to happy faces [31], [53] explained this in terms of enhanced *corr* activity in the baseline interval due to mental preparation for the upcoming stimulus. This may at least partly obscure the subsequent activity in response to angry faces but would enhance the difference to the *corr* response to happy faces.

A further explanation of the present results might interpret the larger *corr* activation observed for anger and sadness, as a linear effect of valence rather than facial mimicry or effort. Thus Larsen and colleagues [23] reported that valence ratings of affective pictures, words and sounds were negatively correlated with *corr* activity. That is, decreasing *corr* activity was accompanied by more positive valence ratings. Hence, if the *corr* activity reflects affective states, we may assume that its response to facial emotional stimuli is also affective in nature and not effort-related. Moreover, Kappas, Lückman, Pleser and Küster [54] showed that valence-related *corr* responses depend on the task, being more pronounced during affective versus complexity ratings.

The distinction between effort-related *corr* activity, emotion-specific facial mimicry, and valence sensitive *corr*-responses needs to be investigated more carefully. In addition, the amount of facial mimicry is influenced by the social context and characteristics of the sender and the perceiver and their interaction intentions [22]. It is an open and intriguing question whether the rank order of individuals concerning facial responses to emotional expressions would change when relevant social information were incorporated into the tasks. Thus, future research might include different context information and subjectively experienced emotional states, for example, by requiring affective valence ratings.

An important limitation in the present study is that expected emotion effects in the *zyg* could not be confirmed. One reason could be lower degree of voluntary control over the *corr* than the *zyg* muscle [55], which could lead to more pronounced incidental facial reactions to emotional expressions in *corr* as compared to *zyg*. Although effects of facial mimicry in *zyg* have been reported during emotional intensity ratings [12] or even unconscious (subliminal) processing [31] others did not find such clear effects [56], [57]. One could speculate that providing contextual information might reveal the expected effects in response to happy expressions (e.g. [58], [22]. Mimicry effects in the *zyg* might also specifically be suppressed by the experimental context like movement restrictions, monotony, and artificiality. More natural settings with real interaction partners might lead to stronger mimicry reactions, especially in response to happy facial expressions [59].

Our results show, for the first time, that reliable measurement of individual differences in incidental facial responses to emotional expressions is feasible. In perspective this provides a tool for testing hypotheses concerning emotion processing deficits in special populations like patients with autism [60] or disruptive behavior disorder [61]. Moreover, research trying to elicit meaningful relations facial responses to emotional expressions and other emotion-related outcomes can benefit from the measurement model established here. For example, automatic mimicking of emotional states as a component of empathy [62] could be examined in a more elaborate way. As a first step, we showed here that under well-controlled conditions, incidental emotion-related *corr* responses are measurable and related to emotion perception. This might be a first piece of evidence from an individual differences perspective on the role of emotion-related facial responses in emotion perception within the scope of embodied simulation theory.

## Supporting Information

Figure S1 Examples of stimuli used for the task *“Identification of Emotion Expressions from Composite Faces” (task 4)*.
(PDF)

Figure S2 Schematic representation of a task trial – *“Visual Search for Faces with Corresponding Emotion Expressions of Different Intensity” (task 6)*.
(PDF)

Table S1 Means and standard deviations of accuracy rates for experimental conditions in all subsamples.
(PDF)

Table S2 Means, standard deviations and standard errors for all experimental conditions and time windows for *corr* and *zyg*.
(PDF)

Table S3 Standardized factor loadings of measurement model 3b.
(PDF)

Table S4 Standardized factor loadings of measurement model of face perception and nested emotion perception.
(PDF)
