# Supplementary material for: Facial EMG Responses to Emotional Expressions Are Related to Emotion Perception Ability
Source: PLoS One. 2014 Jan 28;9(1):e84053. doi: 10.1371/journal.pone.0084053 (PMC3904816; doi:10.1371/journal.pone.0084053)

Panel A: Anger – Disgust, Happiness, Surprise / Identify TOP

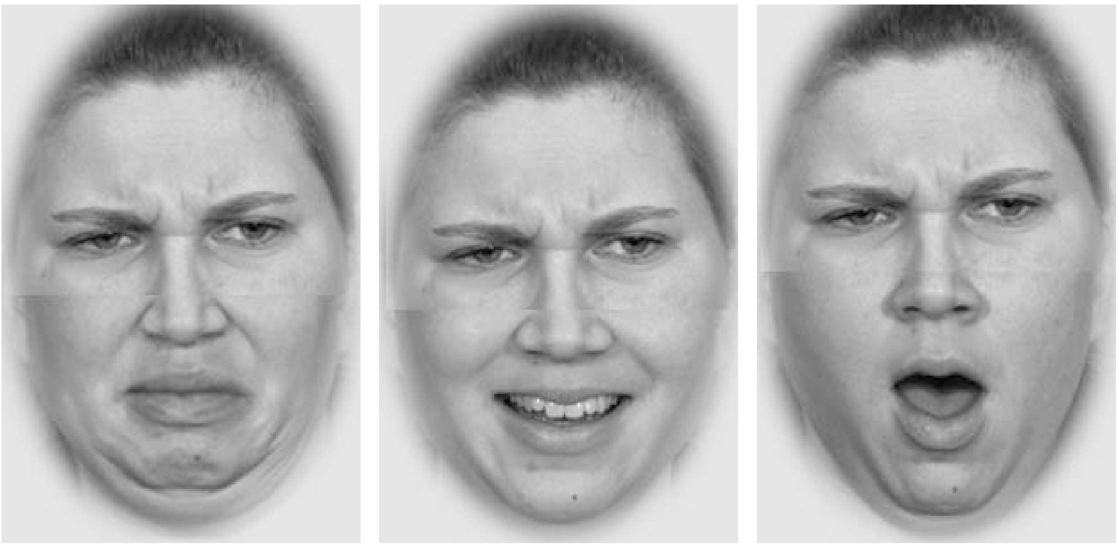

Panel B: Fear – Disgust, Happiness, Surprise / Identify TOP

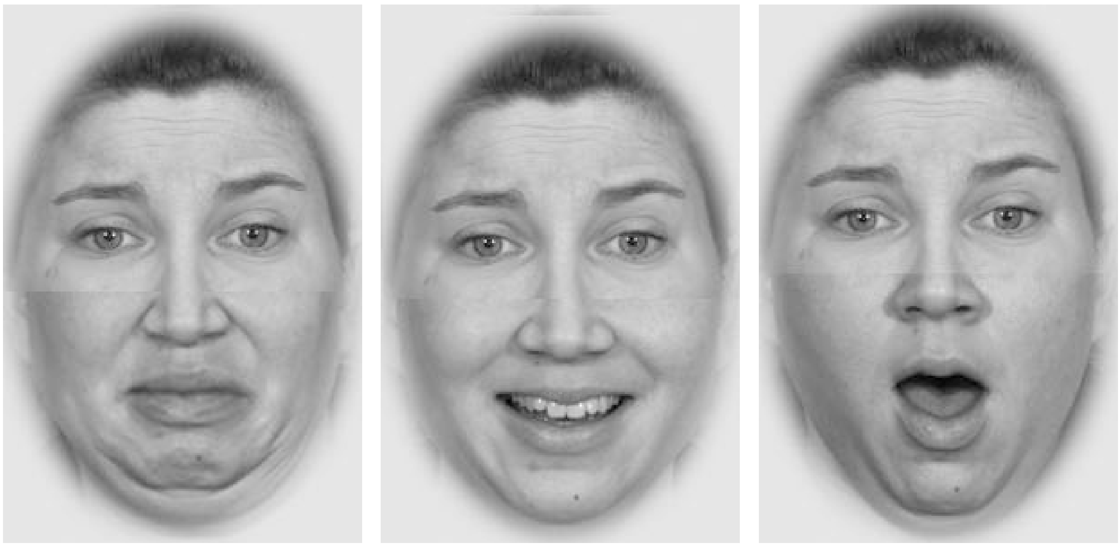

Panel C: Sadness – Disgust, Happiness, Surprise / Identify TOP

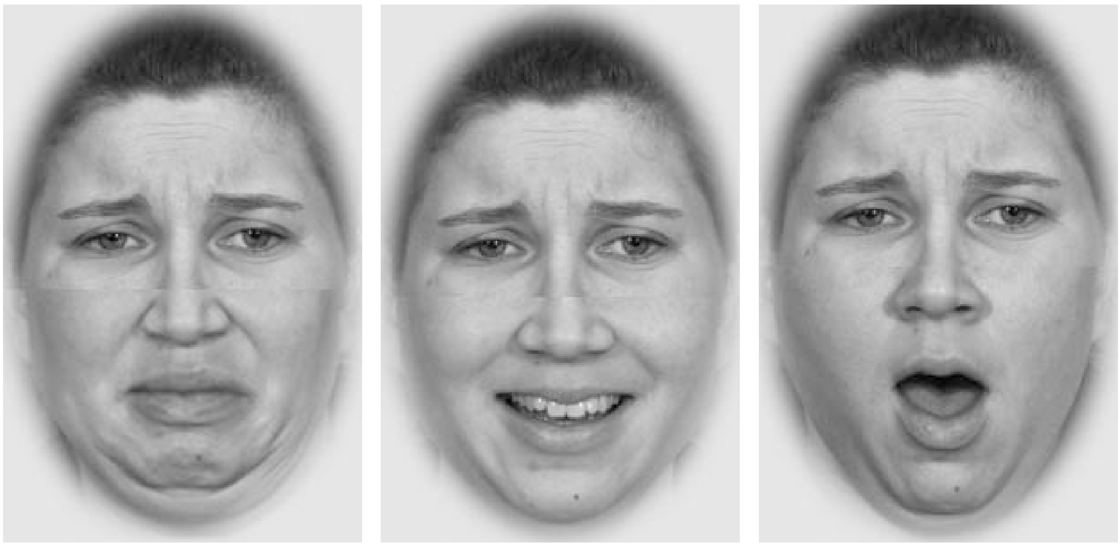

Panel D: Anger, Fear, Sadness – Disgust / Identify BOTTOM

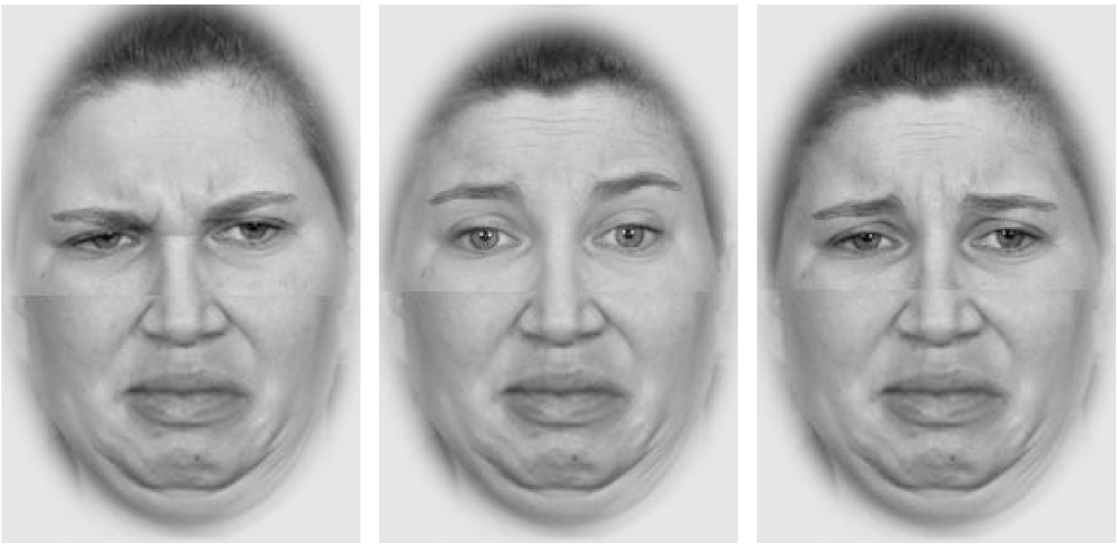

Panel E: Anger, Fear, Sadness – Happiness / Identify BOTTOM

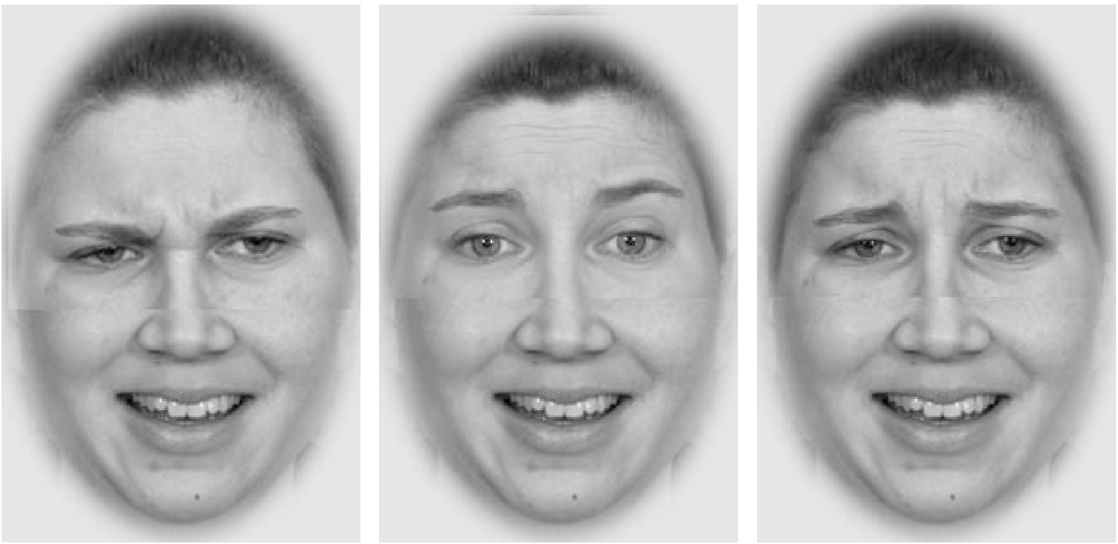

Panel F: Anger, Fear, Sadness – Surprise / Identify BOTTOM

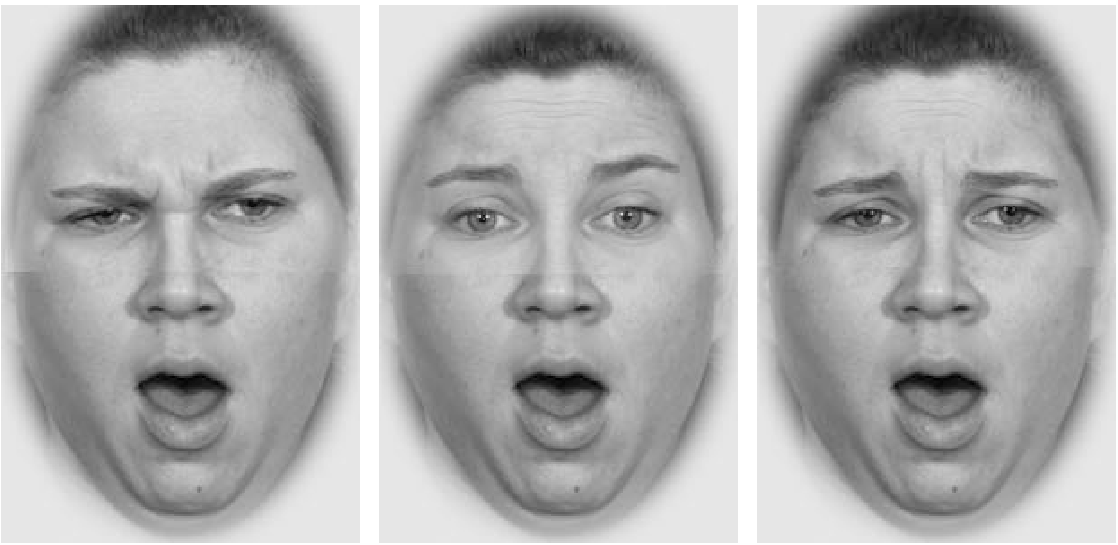

Supplement: Figure S1 — Examples of stimuli used for the task “Identification of Emotion Expressions from Composite Faces” (task 4). (PDF) [file pone.0084053.s001.pdf]
