# Supplementary material for: Facial EMG Responses to Emotional Expressions Are Related to Emotion Perception Ability
Source: PLoS One. 2014 Jan 28;9(1):e84053. doi: 10.1371/journal.pone.0084053 (PMC3904816; doi:10.1371/journal.pone.0084053)
Supplement: Figure S2 — Schematic representation of a task trial – “Visual Search for Faces with Corresponding Emotion Expressions of Different Intensity” (task 6). (PDF) [file pone.0084053.s002.pdf]

Please find 3 expressions that are not identical in their affective content with the prevailing emotional expression!

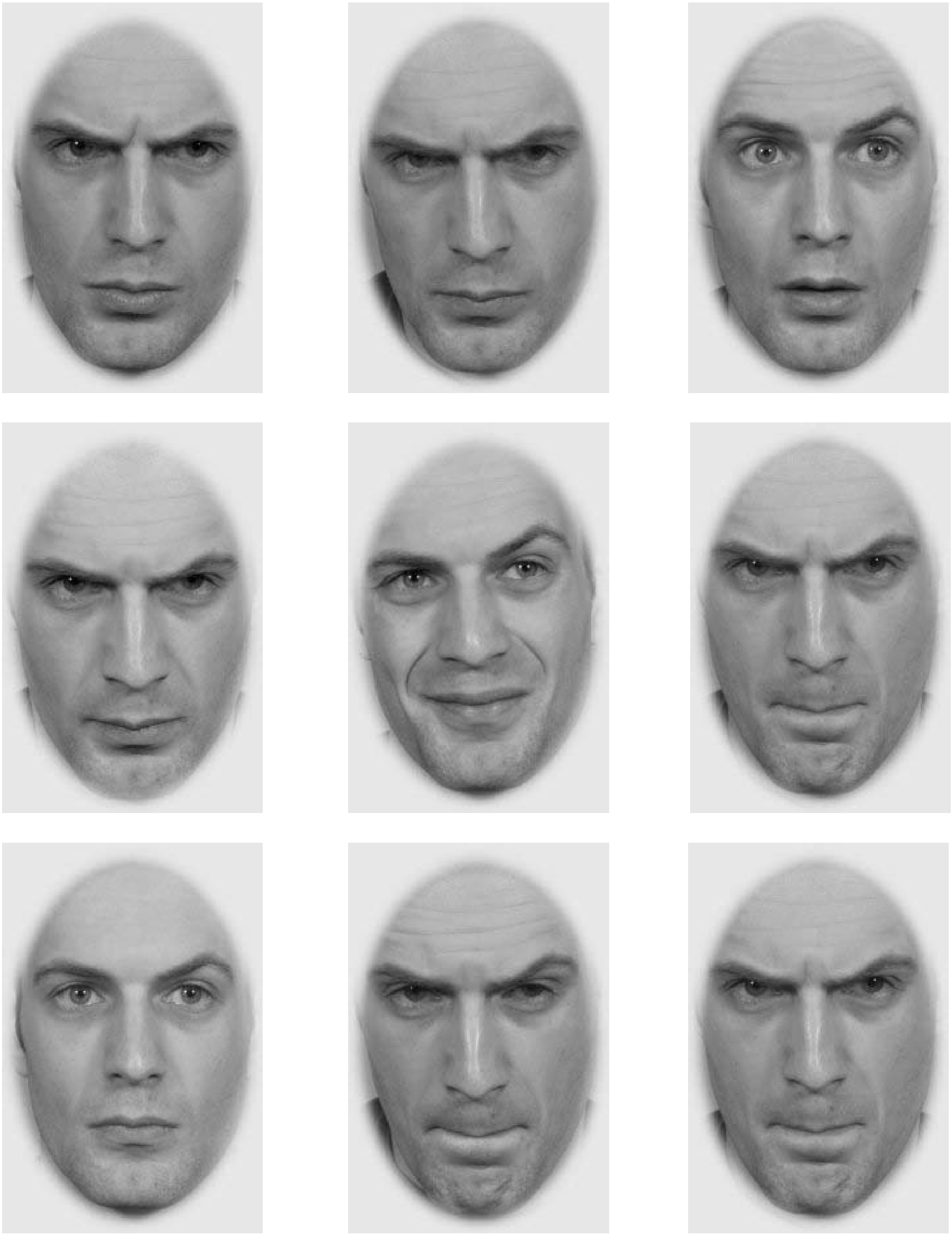

The subjects displayed in the stimuli have given written informed consent to publication of their photographs.
