# Supplementary material for: Facial EMG Responses to Emotional Expressions Are Related to Emotion Perception Ability
Source: PLoS One. 2014 Jan 28;9(1):e84053. doi: 10.1371/journal.pone.0084053 (PMC3904816; doi:10.1371/journal.pone.0084053)
Supplement: Table S1 — Means and standard deviations of accuracy rates for experimental conditions in all subsamples. (PDF) [file pone.0084053.s003.pdf]

Detailed Results

Table S3. Means and standard deviations of accuracy rates for experimental conditions in all subsamples

		all (*N*=110)	*corr* (N_1=94)	*zyg* (N_3=69)
Emotion	Intensity	*M* (*SD*)	*M* (*SD*)	*M* (*SD*)
Anger	80%	.86 (.11)	.87 (.09)	.87 (.09)
	100%	.91 (.09)	.91 (.08)	.91 (.08)
Disgust	80%	.84 (.14)	.85 (.13)	.84 (.13)
	100%	.84 (.15)	.84 (.14)	.83 (.14)
Fear	80%	.75 (.18)	.78 (.16)	.77 (.14)
	100%	.78 (.18)	.80 (.15)	.79 (.14)
Happiness	80%	.99 (.02)	.99 (.02)	.99 (.01)
	100%	.99 (.02)	.99 (.02)	.99 (.01)
Neutral	Chewing	.93 (.17)	.96 (.11)	.97 (.07)
	Blinking	.96 (.06)	.97 (.05)	.96 (.05)
Sadness	80%	.86 (.13)	.87 (.11)	.86 (.11)
	100%	.87 (.14)	.88 (.12)	.88 (.13)
Surprise	80%	.90 (.11)	.91 (.08)	.92 (.08)
	100%	.89 (.12)	.91 (.09)	.91 (.09)
