# Supplementary material for: Facial EMG Responses to Emotional Expressions Are Related to Emotion Perception Ability
Source: PLoS One. 2014 Jan 28;9(1):e84053. doi: 10.1371/journal.pone.0084053 (PMC3904816; doi:10.1371/journal.pone.0084053)
Supplement: Table S2 — Means, standard deviations and standard errors for all experimental conditions and time windows for corr and zyg. (PDF) [file pone.0084053.s004.pdf]

Table S4. Means, standard deviations and standard errors for all experimental conditions and time windows for *corr* and *zyg*

		**0-100**		**100-200**		**200-300**		**300-400**		**400-500**		**500-600**		**600-700**		**700-800**		**800-900**		**900-1000**	
Muscle	Emotion	*M (SD)*	*SE*	*M (SD)*	*SE*	*M (SD)*	*SE*	*M (SD)*	*SE*	*M (SD)*	*SE*	*M (SD)*	*SE*	*M (SD)*	*SE*	*M (SD)*	*SE*	*M (SD)*	*SE*	*M (SD)*	*SE*
Corr	Anger	-.01(.07)	.01	.01(.09)	.01	.05(.15)	.02	.07(.22)	.02	.07(.27)	.03	.08(.29)	.03	.05(.28)	.03	.02(.29)	.03	-.02(.32)	.03	-.02(.32)	.03
	Disgust	.00(.07)	.01	.03(.11)	.01	.05(.15)	.02	.02(.20)	.02	-.02(.23)	.02	-.03(.26)	.03	-.04(.29)	.03	-.07(.30)	.03	-.10(.31)	.03	-.10(.32)	.03
	Fear	.00(.08)	.01	.01(.11)	.01	.03(.14)	.01	.01(.18)	.02	-.03(.21)	.02	-.04(.23)	.02	-.05(.25)	.03	-.07(.26)	.03	-.09(.28)	.03	-.10(.29)	.03
	Happiness	.01(.08)	.01	.03(.10)	.01	-.01(.16)	.02	-.18(.27)	.03	-.31(.38)	.04	-.31(.38)	.04	-.29(.35)	.04	-.25(.33)	.03	-.24(.33)	.03	-.20(.32)	.03
	Neutral	-.01(.07)	.01	.00(.10)	.01	.03(.13)	.01	.03(.17)	.02	.03(.19)	.02	.00(.20)	.02	-.04(.23)	.02	-.09(.26)	.03	-.13(.29)	.03	-.13(.29)	.03
	Sadness	.00(.08)	.01	.03(.11)	.01	.05(.14)	.01	.08(.18)	.02	.07(.21)	.02	.07(.23)	.02	.05(.24)	.02	.03(.24)	.03	-.01(.26)	.03	-.02(.27)	.03
	Surprise	-.01(.07)	.01	.01(.10)	.01	.04(.15)	.02	.02(.18)	.02	-.02(.21)	.02	-.05(.24)	.03	-.07(.27)	.03	-.09(.28)	.03	-.12(.29)	.03	-.14(.30)	.03
Zyg	Anger	-.01(.04)	.00	-.02(.05)	.01	-.02(.07)	.01	-.02(.08)	.01	-.02(.08)	.01	-.03(.09)	.01	-.04(.10)	.01	.06(.10)	.01	-.06(.10)	.01	-.06(.11)	.01
	Disgust	-.01(.03)	.00	.00(.05)	.01	.00(.06)	.01	.00(.06)	.01	-.01(.06)	.01	-.01(.07)	.01	-.02(.08)	.01	-.03(.09)	.01	-.04(.09)	.01	-.04(.09)	.01
	Fear	-.01(.03)	.00	-.01(.05)	.01	-.01(.07)	.01	-.01(.08)	.01	-.01(.09)	.01	-.01(.09)	.01	-.02(.10)	.01	-.03(.10)	.01	-.03(.11)	.01	-.03(.11)	.01
	Happiness	-.01(.03)	.00	.00(.05)	.01	.00(.05)	.01	.00(.06)	.01	-.01(.06)	.01	-.01 (.07)	.01	-.03(.08)	.01	-.04(.10)	.01	-.04(.10)	.01	-.04(.10)	.01
	Neutral	-.01(.03)	.00	-.01(.05)	.01	-.01(.06)	.01	-.01(.07)	.01	-.01(.07)	.01	-.01(.08)	.01	-.02(.09)	.01	-.04(.09)	.01	-.04(.09)	.01	-.03(.09)	.01
	Sadness	-.01(.04)	.00	-.01(.05)	.01	-.02 (.07)	.01	-.02 (.07)	.01	-.01(.07)	.01	-.01(.08)	.01	-.01(.09)	.01	-.03(.10)	.01	-.03(.11)	.01	-.02(.11)	.01
	Surprise	-.01(.03)	.00	-.01(.04)	.01	-.02 (.07)	.01	-.02 (.07)	.01	-.03(.06)	.01	-.02(.07)	.01	-.03(.08)	.01	-.04(.08)	.01	-.04(.08)	.01	-.04(.08)	.01
