# Supplementary material for: Facial EMG Responses to Emotional Expressions Are Related to Emotion Perception Ability
Source: PLoS One. 2014 Jan 28;9(1):e84053. doi: 10.1371/journal.pone.0084053 (PMC3904816; doi:10.1371/journal.pone.0084053)
Supplement: Table S3 — Standardized factor loadings of measurement model 3b. (PDF) [file pone.0084053.s005.pdf]

Table S5. Standardized factor loadings of measurement model 3b

factor	Fcorr															
indicator	ne1	ne2	ne3	ne4	an1	an2	an3	an4	ha1	ha2	ha3	ha4	sa1	sa2	sa3	sa4
standardized loading	.647*	.665*	.620*	.655*	.745*	.739*	.707*	.781*	.768*	.764*	.794*	.830*	.582*	.640*	.543*	.513*
factor	ANcorr				HAcorr				SAcorr							
indicator	an1	an2	an3	an4	ha1	ha2	ha3	ha4	sa1	sa2	sa3	sa4				
standardized loading	.558*	.566*	517*	.535*	.682*	.615*	.763*	.757*	.168	.336	.562	.265				

*$p < .05$, two-tailed.
