# Supplementary material for: Facial EMG Responses to Emotional Expressions Are Related to Emotion Perception Ability
Source: PLoS One. 2014 Jan 28;9(1):e84053. doi: 10.1371/journal.pone.0084053 (PMC3904816; doi:10.1371/journal.pone.0084053)
Supplement: Table S4 — Standardized factor loadings of measurement model of face perception and nested emotion perception. (PDF) [file pone.0084053.s006.pdf]

Table S6. Standardized factor loadings of measurement model of face perception and nested emotion perception

factor	FP								EP		
indicator	face1	face2	face3	face4	face5	emo1	emo2	emo3	emo1	emo2	emo3
standardized loading	.597*	.501*	.479*	.734*	.642*	.438*	.413*	.516*	.311*	.350*	.461*

*p < .05, two-tailed.
